# Toward a Climate of Scientific Integrity

**DOI:** 10.1371/journal.pbio.0040084

**Published:** 2006-03-14

**Authors:** Beth A Fischer, Michael J Zigmond

## Abstract

A review of the new edition of Macrina's "Scientific integrity: Text and cases in responsible conduct of research" highlights the continuing need for teaching ethical guidelines to young researchers.

Periodically the media is filled with articles about scientific misconduct—fabricated data, misrepresented protocols, and questionable authorship. Are these incidents anomalies or indicators of inadequate attention being paid to creating a climate of integrity? We do not know. However, it is clear to us that there is much we can do to improve the climate in which we do our science. In this regard, *Scientific Integrity* is an important tool.

In the ten years since publication of the first edition, *Scientific Integrity* has become an indispensable text on the responsible conduct of research (RCR). Written by Francis Macrina, Professor of Microbiology and Immunology and Director of the Philips Institute at Virginia Commonwealth University, and now in its third edition, this book reflects the knowledge and perspectives of an individual who is both an accomplished scientist and an educator. Anyone who conducts research, trains researchers, or is otherwise interested in the practice of science is likely to benefit from reading *Scientific Integrity*.

Macrina assumes little prior knowledge on the part of the reader, and provides a comprehensive and engaging introduction to what some might otherwise perceive as a dry subject. The content is tailored to the needs of practitioners of science, and covers the nine core RCR topics that the United States Public Health Service recommends including in RCR training: (1) data acquisition, management, sharing, and ownership, (2) mentor/trainee responsibilities, (3) publication practices and responsible authorship, (4) peer review, (5) collaborative science, (6) research misconduct, (7) conflict of interest and commitment, and (8) the use of humans and (9) animals as research subjects [1].

The book is well organized. Two initial chapters introduce the concept of responsible conduct and its philosophical underpinnings. These are followed by chapters that cover the core issues in RCR. Each contains a set of features that are useful for students and instructors alike: a list of discussion questions and a number of brief case studies in which the reader is presented with an ethical dilemma and asked to respond. Also included in each chapter is a select list of resources that Macrina has identified for readers as the essentials to start with. Through the book's Web site (http://www.scientificintegrity.net), readers will be able to access any updated or new links to resources and reference material.

Case discussions make a valuable contribution to RCR training as they require students to apply what they are learning to a specific problem. This helps students process and integrate new information, as well as develop their analytical and reasoning skills. This type of active, rather than passive, interaction with the material is a standard principle used to promote adult learning, and is advocated by the Institute of Medicine for use in providing RCR instruction [2].

Although many books on RCR include cases for discussion, the cases are often unrealistic and/or too simplistic. Not so in *Scientific Integrity*. There is ample fodder for generating good classroom discussions, and experienced scientists are likely to identify with some portions of the scenarios—based on either their own experiences or those of their colleagues.

The appendices also make a substantial contribution to the book. There, readers will find samples of completed applications for patents and for the use of human and animal subjects. And for instructors, there is a play that can be staged in class and then discussed. In addition, Macrina includes a form that instructors can use to survey their students' knowledge of RCR, thereby enabling them to better address their students' needs.

The third edition of *Scientific Integrity* includes information on some new topics—concerns that have emerged alongside the development of technologies, such as electronic record keeping, open-access publishing, and bioterrorism. These are valuable additions to the book's content. Moreover, we feel that these also serve to illustrate an important principle in which Macrina also believes—that the ethical challenges faced by researchers continually evolve. Thus, there is no single list of rules to memorize that, if followed, will make someone an ethical researcher. Instead, individuals need to continually update their knowledge of acceptable practices, as well as learn how to deal with novel situations for which there are no rules as of yet [3]. Macrina provides a brief overview of one such approach.

Although the cases are one of the book's strengths, they also reflect a shortcoming—a lack of attention to issues of diversity. The author might be surprised to learn the results of our analysis—that characters in the scenarios are twice as likely to be male as female, and when women are included in the cases, they frequently hold positions that are subordinate in rank. (Interestingly, all four of the quotes on the back cover of the book were provided by men, including one of us [MZ].) In addition, last names were for the most part typical of Anglo-Americans; few if any names reflected Asian, Hispanic, or Middle Eastern origins. We hope these oversights are addressed in the Fourth edition.

Our other criticism of the text is that the author and other contributors do not provide readers with notes on the case studies indicating the major ethical concerns, as are sometimes provided in the back of textbooks. Whereas we understand Macrina's desire to leave the door open for multiple acceptable solutions to a problem, we disagree with the other rationale he provides for the omission, namely, that he (and the other contributors) “believe that ‘wrong answers’ will be obvious” (p. xxii). It would be great if this was so—we would no longer need to provide instruction in RCR!

But our most serious concern is not with *Scientific Integrity* itself but with the limited ways in which we expect it will be used. Almost certainly, the great majority of readers will be graduate students in required seminars on RCR. Indeed, a quick search of syllabi posted on the internet reveals that *Scientific Integrity* is the primary text in numerous RCR courses around the US. This is good, but it is far from sufficient.

There are two problems. First, graduate students represent only a fraction of those engaged in the research enterprise. There are also faculty, staff, postdoctoral fellows, and a whole world of people carrying out their research outside of academic institutions. All of them should also be obtaining RCR instruction. This has been recognized many times. In 1995, the Commission on Research Integrity, established by the US Department of Health and Human Services and chaired by Kenneth J. Ryan, issued its report. Many involved in research disagreed with some of the commission's conclusions, but it is hard to disagree with their proposal to “expand existing institutional assurances to require that research institutions provide research integrity education for *all individuals supported by PHS research funds ”* (italics added) [4]. Again, in 2000 the US Public Health Service developed a policy that would “require research institutions to provide training in the responsible conduct of research to *all staff engaged in research or research training with PHS funds* ” (italics added) [5]. That policy was suspended, and to our knowledge those recommendations remain on the shelf. Yet, unfortunately, experience tells us that it is only when RCR instruction is mandated, as it was in 1989 for training grants funded by the National Institutes of Health [6], that it becomes commonplace. Thus, we hope the recommendations will soon be resurrected and approved.

***Good* research is *responsible* research.**

Second, whereas a one-time course or seminar can be extremely valuable, effective RCR training must include much more. No one learns all they need to know about a complex subject from a single course. Active interaction with the material and exposure over time are required. Why would learning responsible conduct be different? Even more importantly, we believe that for RCR to become integrated into the fabric of research, it cannot remain as a separate entity but must be integrated into all other aspects of training and performing research—core courses should include discussions of the ethical dimensions of the material being presented, lab meetings should include a discussion of ethical issues that pertain to the day-to-day activities of the group, and individuals presenting their work in seminars, in symposia, and in print should comment on ethical issues of relevance to their research [3]. Only in this way will it become clear that responsible conduct is an integral part of doing research. *Scientific Integrity* can serve as a valuable resource in this endeavor and should be read and discussed by anyone involved in research, regardless of their position. For it is only by integrating discussions of RCR into all aspects of research that we can expect practitioners to develop a deep understanding of the truism that *good* research is *responsible* research.

**Figure:**
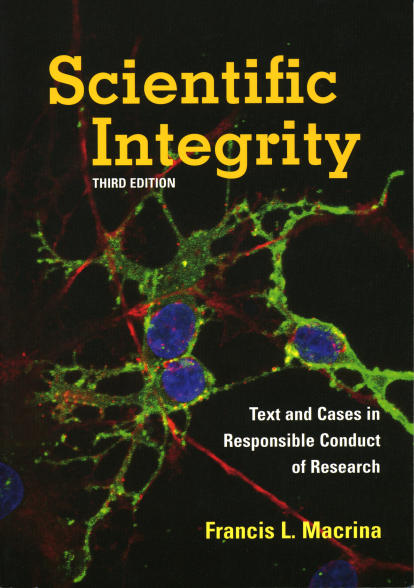


## Abbreviation

RCR: responsible conduct of research
